# Level of Anxiety, Depression, Self-Esteem, Social Anxiety, and Quality of Life among the Women with Polycystic Ovary Syndrome

**DOI:** 10.1155/2013/851815

**Published:** 2013-07-09

**Authors:** Gökhan Açmaz, Evrim Albayrak, Banu Acmaz, Mürüvvet Başer, Murat Soyak, Gökmen Zararsız, İptisam İpekMüderris

**Affiliations:** ^1^Department of Obstetrics and Gynecology, Kayseri Education and Research Hospital, Kayseri, Turkey; ^2^Department of Health Sciences, Erciyes University, School of Medicine, Kayseri, Turkey; ^3^Department of Internal Medicine, Erciyes University, School of Medicine, Kayseri, Turkey; ^4^Department of Psychiatry, Kayseri Education and Research Hospital, Kayseri, Turkey; ^5^Department of Statistics, Erciyes University, School of Medicine, Kayseri, Turkey; ^6^Department of Obstetrics and Gynecology, Erciyes University, School of Medicine, Kayseri, Turkey

## Abstract

*Introduction*. Polycystic ovary syndrome (PCOS) is a heterogeneous disease and many symptoms are seen with varying degrees. The aim of the present study was to determine which symptoms increased such problems as depression, anxiety, low self-esteem, and social worry by classifying PCOS according to symptoms. *Methods*. The study was carried out with two groups. The first group consisted of 86 patients who were diagnosed with PCOS and the second group consisted of 47 healthy volunteers. Liebowitz' Social Anxiety Scale, Rosenberg' Self-Esteem Scale, Short-Form 36, Quality of Life Scale, Beck Anxiety Inventory, and Beck Depression Inventory were administered to each volunteer. *Results*. Depression scores of infertile group were higher while anxiety scores of the obese group were bigger than other groups. It was the obesity group that received the smallest score in self-esteem and trust in people and the highest score in sensitiveness to criticism. The most affected group was oligomenorrhea-hirsutism group in terms of physical functioning, physical role function, pain, social functioning, emotional role function, and emotional well-being. *Conclusion*. We suggest that not only gynecologist but also a multidisciplinary team may examine these patients.

## 1. Introduction

Polycystic ovary syndrome (PCOS) is a commonly seen endocrinologic disorder at reproductive age. Classical symptoms of the disorder which affects 10% of all women are irregular menstrual bleeding, clinical or biochemical hyperandrogenemia, and polycystic ovary syndrome appearance in ultrasonography [1]. In case of the presence of the two above-mentioned symptoms, PCOS diagnosis is made [2].

PCOS has a rather heterogeneous patient group, and many symptoms are seen with varying degrees. In the study of Vural et al. [3], there was no difference between the control group and study group in terms of body mass index, whereas Gambineri et al. [4] argued that one of the most common symptoms of PCOS was obesity, and obesity is present with a prevalence of 50%. Hirsutism and acne are the important symptoms of PCOS, and these symptoms may be the consequence of either androgen excess or increased sensitivity to androgens receptors in spite of a normal level of androgen [5]. These symptoms affect the patients psychosocially—in particular—and may cause them to keep away from the society. Particularly among the adolescent girls, it may lead to anxiety and depression as a result of the changing body perception due to the fear that they are not liked [6, 7]. 

In the literature, we did not find any study that investigated social anxiety and avoidance among PCOS patients. It is important to mention that ours is the first one.

One of the characteristics of PCOS is infertility and/or subfertility. Duration of infertility, duration of its treatment, types of treatment, and causes of infertility among the women who wish to have child create pressure on the couples. Also, child expectation of the society and expressing this expectation affect the depth and severity of psychiatric symptoms [8]. However, the relevant literature is rather limited and contradictory [9]. A meta-analysis published reported that depression frequency increases with PCOS. According to this meta-analysis, when previously published reviews and meta-analysis were examined, it was seen that almost all of these studies emphasized that the number of the groups was small and they discussed PCOS as a whole but did not deal with its symptoms, and therefore, the concept of causality was lost [10].

The aim of the present study was to determine which symptoms increased such problems as depression, anxiety, low self-esteem, and social worry by classifying PCOS according to symptoms and to help the treatment-to-be-used for PCOS be individualized by exploring how much quality of life of women was affected by these problems experienced by women. 

## 2. Method

This study was conducted at Kayseri Education and Research Hospital of Medicine. The study was approved by the Ethical Committee of University Hospital, and written informed consent was obtained from all the participants. 

### 2.1. Participants

The study was carried out with two groups. The first group consisted of 86 patients who were diagnosed with PCOS according to 2003 Rotterdam Criteria, had no physical disease but PCOS, did not receive any treatment (before the treatment) for PCOS and had at least primary school degree. The second group consisted of 47 healthy volunteer participants in reproductive age. Those who had thyroid disorders, DM, Cushing' disease, positive malignancy, congenital adrenal hyperplasia, psychotic disorders and used antidepressants or steroidal hormone drugs and mood stabilizers (Li, Valproic acid, etc.) were excluded from the study. 

Patients with PCOS were classified according to the complaints at the time of polyclinic admission. Patients who went to the polyclinic to have child but were diagnosed with PCOS made up *infertility* group; patients who went to the polyclinic due to excess facial and body hair (hirsutism), acne and irregular menstrual cycles but were diagnosed with PCOS made up *oligomenorrhea-hirsutism *group and patients who could not lose had weight and had a BMI ≥ 30 but were diagnosed with PCOS constituted *overweight-obesity* group.

### 2.2. Data Collection

Liebowitz' Social Anxiety Scale (LSAS), Rosenberg' Self-Esteem Scale (RSES), Short-Form 36 (SF-36) Quality of Life Scale, Beck Anxiety Inventory (BAI), and Beck Depression Inventory (BDI) were administered to each of the participant women. These scales and inventories were used and scored by the same psychiatrist. The Structured Clinical Interview for DSM-IV Axis I Disorders (SCID-I) and the Structured Clinical Interview for DSM-IV Axis II Disorders (SCID-II) were administered and examined by the same psychiatrist, too. As a result of the examination, those who had personality disorders were not included in the study. Sociodemographic form and clinical information form were administered to the patients in PCOS group and to those in the healthy group, too. The form addressed such sociodemographic characteristics as age, educational level, income status and income perception, marital status, profession, and intention to have child. 

The data collection phase of the study took six months.

### 2.3. Data Collection Instruments

#### 2.3.1. Liebowitz' Social Anxiety Scale (LSAS)

 It is a Likert-type self-rated scale with 24 questions. It measures anxiety and avoidance in case of various social situations. There are two subscales that measure the severity of anxiety and avoidance experienced in different social settings. Score to be obtained from the each subscale ranges from 0 to 72 and total score of the scale changes between 0 and 144. Higher scores indicate more severe social anxiety and avoidance. The advised cutoff point is 25 for each subscale and 50 for total score. The scale was developed by Liebowitz [11], and validity and reliability tests of the Turkish form were performed by Soykan et al. [12].

#### 2.3.2. Rosenberg' Self-Esteem Scale (RSES)

 It is a Likert-type self-rated scale composed of 10 questions and was designed by Rosenberg [13]. Validity and reliability tests of the Turkish form were performed by Çuhadaroğlu [14]. The first ten items of the scale are used to evaluate self-esteem. A total score between 0 and 1 indicates high level of self-esteem, a total score between 2 and 4 indicates moderate level of self-esteem and a total score between 5 and 6 points out low level of self-esteem [14]. 

#### 2.3.3. SF-36 Quality of Life Scale

SF-36 is the most widely used scale in order to measure the quality of life. It consists of 36 questions. The scale was designed by Rand Corporation in 1992 and was put into service [15]. Validity and reliability tests of the Turkish version were performed by Koçyiğit et al. [16]. 

#### 2.3.4. Beck Depression Inventory (BDI)

The inventory was developed by Beck et al. [17] and measures physical, emotional, and mental symptoms in depression. It is self-rated inventory with 21-symptom category. Higher total scores indicate higher severity of depression. Validity and reliability tests of the Turkish version were performed by Hisli [18].

#### 2.3.5. Beck Anxiety Inventory (BAI)

 It is self-rated inventory used to determine the frequency of anxiety symptoms. It is composed of 21 items and is a Likert-type inventory rated from 0 to 3. Higher total scores indicate higher severity of anxiety [19]. Validity and reliability tests of the Turkish version were performed by Ulusoy [20]. 

### 2.4. Data Analysis

The analysis of the data was performed with R 2.15.0 software. Shapiro-Wilk test was used in order to determine whether or not the data followed a normal distribution. As for variance homogeneity, Levene' test was used. The data were presented as frequency and percentages, means ± SD or median (25th and 75th). For the qualitative data, Pearson' chi-square analysis was performed for intergroup comparisons while for the quantitative data one-way variance analysis, Kruskal-Wallis *H* test and Welch test were used. For the multicomparisons, Bonferroni' corrected chi-square analyses, *t*-test, and Mann-Whitney *U* test were used. For all analyses, statistical significance was defined by a probability level of *P* < 0.05.

## 3. Results

Table 1 included the data related to women's age, marital status, educational status, profession, income and income perception, and clinical signs of PCOS. It was seen that groups were similar in terms of age, educational status, profession, income and income perception, and living residence (*P* > 0.05). However, parameters of PCOS group were different as compared with healthy group in terms of marital status, intention to have child and the number of the children, and classic PCOS signs such as acne, hirsutism, oligomenorrhea, and obesity (*P* < 0.05).

Table 2 included anxiety and avoidance scores of BDI, BAI and LSAS. When the results were investigated, depression scores of infertile group were higher while anxiety scores of the obese group were bigger than other groups (*P* < 0.001). The groups of the obese and oligomenorrhea-hirsutism received higher scores in anxiety and avoidance as compared with the healthy group (*P* < 0.001). 

Table 3 included the data related to self-esteem (SE), continuity in self-concepts (CSC), trust in people (TIP), sensitiveness to criticism (SC), depressive affect (DA), daydreaming (DD), psychosomatic symptoms (PSS), feeling threat in interpersonal relations (FTIR), intensity of discussion (ID), parental interest (PI), father-relation (FR), and psychic isolation (PsI). It was seen that it was the obesity group that received the smallest score in SE (62.1%) and TIP (44.8%) and the highest score in SC (75.9) (*P* < 0.001). It was infertile group that received the biggest score in DA (50.0%) and DD (68.2%) (*P* < 0.001). The scores of PSS and PsI were found to be considerably higher in the infertile group and the obese group (*P* < 0.001). There was no significant difference in other parameters. 

Table 4 included mean scores related to physical functioning (PF), physical role function (PRF), pain, general health (GH), vitality, social functioning (SF), emotional role function (ERF), and emotional well-being (EWB). Although there were significant differences in all of these parameters compared to the healthy group, the most affected group was oligomenorrhea-hirsutism group in terms of PF, PRF, pain, SF, ERF, and EWB, but it was obesity group that was affected by GH most and it was infertility group that was affected by vitality most (*P* < 0.001).

## 4. Discussion

Recently, psychiatric morbidity in PCOS has been the research focus and an important study topic. Obesity, hirsutism, and infertility are important signs of PCOS. These signs make patients with PCOS feel different and less feminine as compared with the society, which may result in some psychiatric problems [21]. In the study, patients were sorted into groups in terms of their complaints because PCOS is a heterogeneous disease, and these groups were compared to each other and to a healthy group. In the literature, patients were generally examined only in PCOS without being classified into groups and the scales and inventories were administered without taking their complaints in consideration. 

The study of Jedel et al. compared 30 patients with PCOS to 30 healthy volunteer, and found that anxiety increased in patients with PCOS but no difference existed in depression. However, authors found that the number of the children in PCOS group and control group was similar, and therefore infertile patients were not investigated exactly [22]. Hahn pointed out that depressive complaints increased among the patients with PCOS who had small number of children but higher BMI and hirsutism complaints; however, told told that anxiety was similar to those in healthy control group [6]. 

In a study in which 115 infertile women with PCOS were investigated, it was found out that 23.9% of the patients had moderate level of depression while 25.2% had relevant depression. It is revealed that 41% of the patients were composed of those who went to the clinics due to infertility and 72.5% of the patients did not become pregnant despite regular sexual relation for one year [23]. Our study was composed of patients all of whom could not become pregnant despite regular sexual intercourse at least for one year and therefore went to infertility clinics. In this regard, being different from the study of Tan et al., our study investigated completely infertile group and included patients who were infertile for longer periods. Also, it seemed that infertile patients in our study were subjected to social and family pressure for longer periods. It was possible that depression scores of our participant infertile women may have been higher due to these factors. When this study and other three studies [6, 22, 23] were considered, it may be suggested that the actual reason for PCOS was infertility and that depressive complaints would be decreased as the number of the infertile patients reduced. 

Jedel et al. expressed that there was not difference for depression between PCOS and BMI-matched controls but anxiety increased in PCOS. Although both groups were similar in the present study in terms of BMI, their BMI values were under 25 kg/m^2^, which made us conclude that obesity—one of the most important characteristics of PCOS—was not assessed properly [22]. 

Hahn et al. compared patients with PCOS whose BMI values were higher to healthy control participants whose BMI values were normal and found that depression increased among these patients [6]. 

In our study, too, it was infertility group whose BDI score was the highest. Nearly 50% of the patients had high DA and 36.4% had moderate DA. Therefore, consistent findings were obtained from both inventories. In the obese group, it was seen that anxiety existed prominently, depression was higher than healthy control group but was lower than infertile group and hirsutism group, which made us think that the actual determinant of depression was infertility and hirsutism. 

Between our study and literature were similarities in terms of Quality of Life Scale. Hahn et al. administered quality of life scale to the patients of PCOS, and similar results as ours were obtained but no difference was found between the groups in terms of general health [6]. In our study, it was noted that the most affected group was oligomenorrhea-hirsutism group in terms of PF, PRF, pain, SF, ERF, and EWB but it was obesity group that was affected by GH most and it was infertility group that was affected by vitality most. 

In a study in which infertile Muslim and Austrian women who lived in Austria and had PCOS were compared in terms of quality of life, it was detected that Muslim women associated oligomenorrhea periods mostly with infertility, and it was emphasized that this situation affected their quality of life [24]. 

The most affecting factors for the quality of life among women were irregular menstrual cycle and hirsutism. The effect created by irregular menstrual cycle may play a crucial core in patients' perceptions, and therefore, irregular menstrual cycle may sometimes be perceived as the reason of infertility or hirsutism, which results—in turn—in stress in patients, and stress causes impairment of ovulation more severely and aggravates signs. This is a chicken-and-egg situation which deteriorates quality of life more and more. 

 In the literature, we did not find any study that investigated social anxiety and avoidance among PCOS patients. It is important to mention that ours is the first one. It was found out that the most affected groups in terms of anxiety and avoidance were hirsutism-oligomenorrhea and obesity groups. The common characteristic of these two groups was that they were composed of patients who were phenotypically affected. Obesity and hirsutism may lead to higher scores in anxiety and avoidance scales. 

 There was only one paper in which Rosenberg' Self-esteem Scale was administered in PCOS patients. In this paper, it was highlighted that hirsutism and BMI of PCOS patients may particularly be effective upon their negative interaction. The study included no infertile group and the participants were not investigated in relation with the intention to have children [25]. 

In our study, DA scores and DD scores increased among the infertile group most while PSS and PsI considerably increased both in infertile group and obese group. Because the highest score in BDI was obtained by infertility group, findings of the two scales were consistent. Psychosomatic signs are commonly seen among the depressive patients. Therefore, that higher level of psychosomatic signs in infertile group may be attributed to depression experienced by infertile PCOS patients. 

In the literature, patients who were diagnosed with PCOS were generally investigated without considering their clinical characteristics. But, PCOS is a highly heterogeneous disease. Some patients are infertile and are subjected to social pressure due to the importance given to the children by the society. Women in the study group and in healthy control group are composed of young women who belong to reproductive age. Young patients undergo difficulty in not finding a partner caused by the problems due to hirsutism, obesity and suffer from the inability to form a relation with the opposite sex. Therefore, our study took clinical characteristics of the patients into consideration, and groups were created in line with these findings and complaints. There is a need for the new studies to be undertaken using volunteers of the same characteristics but without PCOS. We suggest that not only gynecologist but also a multidisciplinary team may examine these patients. Also, all family members—husbands in particular—should determine domestic social relations in the family. Social support system should be established and provided, and a family-centered approach should be adopted. 

## Figures and Tables

**Table 1:** Basic Characteristics of study and control groups.

Variables	Control (*n* = 47)	Hirsutism-acnea (*n* = 35)	Infertility (*n* = 22)	Obesity (*n* = 29)	*P*
Age	27.77 ± 6.49	26.14 ± 4.98	24.32 ± 4.59	26.00 ± 6.58	0.143
Marital status					
Single	18 (38.3)^a^	15 (42.9)^a^	0 (0.0)^b^	6 (20.7)^a^	0.003
Married	26 (55.3)^ab^	17 (48.6)^b^	22 (100.0)^c^	22 (75.9)^a^	
Divorced	3 (6.4)^a^	3 (8.6)^a^	0 (0.0)^a^	1 (3.4)^a^	
Educational status					
Primary	9 (19.1)	7 (20.0)	6 (27.3)	7 (24.1)	0.689
Secondary	21 (44.7)	17 (48.6)	11 (50.0)	17 (58.6)	
University	17 (36.2)	11 (31.4)	5 (22.7)	5 (17.2)	
Profession					
Housewife	15 (31.9)^a^	13 (37.1)^ab^	14 (63.6)^bc^	18 (62.1)^c^	0.014
Working	32 (68.1)^a^	22 (62.9)^ab^	8 (36.4)^bc^	11 (37.9)^c^	
Income					
≤1000€	12 (25.5)	7 (20.0)	10 (45.5)	10 (34.5)	0.172
1000–2000€	28 (59.6)	27 (77.1)	10 (45.5)	16 (55.2)	
>3000€	7 (14.9)	1 (2.9)	2 (9.0)	3 (10.3)	
Income perception					
Sufficient	22 (46.8)	21 (60.0)	13 (59.1)	16 (55.2)	0.630
Insufficient	25 (53.2)	14 (40.0)	9 (40.9)	13 (44.8)	
Living residence					
Rent	22 (46.8)	20 (57.1)	9 (40.9)	12 (41.4)	0.545
Landlord	25 (53.2)	15 (42.9)	13 (59.1)	17 (58.6)	
Number of childs	1.00 (0.00–2.00)^a^	1.00 (0.00–2.00)^a^	0.00 (0.00-0.00)^b^	1.00 (0.00–2.00)^a^	<0.001
Intention to have child					
Yes	11 (23.4)^a^	10 (28.6)^ab^	22 (100.0)^c^	15 (51.7)^b^	<0.001
No	36 (76.6)^a^	25 (71.4)^ab^	0 (0.0)^c^	14 (48.3)^b^	
Acne					
Yes	3 (6.4)^a^	30 (85.7)^b^	9 (40.9)^c^	16 (55.2)^c^	<0.001
No	44 (93.6)^a^	5 (14.3)^b^	13 (59.1)^c^	13 (44.8)^c^	
Hirsutism					
Yes	0 (0.0)^a^	21 (60.0)^b^	8 (36.4)^b^	11 (37.9)^b^	<0.001
No	47 (100.0)^a^	14 (40.0)^b^	14 (63.6)^b^	18 (62.1)^b^	
Oligomenorrhea					
Yes	0 (0.0)^a^	33 (94.3)^b^	14 (63.6)^c^	9 (31.0)^d^	<0.001
No	47 (100.0)^a^	2 (5.7)^b^	8 (36.4)^c^	20 (69.0)^d^	
BMI (kg/m^2^)	23.37 ± 3.13^a^	24.45 ± 2.75^a^	24.35 ± 3.48^a^	33.59 ± 2.61^b^	<0.001

Values expressed as *n* (%), mean ± SD, or median (25th–75th percentiles) groups with different superscript letters were found to have statistically significant differences.

**Table 2:** Evaluation of BDI, BAI, and LSAS in PCOS and control groups.

Variables	Control (*n* = 47)	Hirsutism-acnea (*n* = 35)	Infertility (*n* = 22)	Obesity (*n* = 29)	*P*
BDI	12.28 ± 6.35^a^	24.46 ± 9.76^bd^	30.59 ± 11.31^bc^	19.10 ± 8.52^d^	<0.001
BAI	12.00 (9.00–16.00)^a^	20.00 (14.00–26.00)^bc^	13.50 (10.00–21.00)^ac^	24.00 (21.00–34.00)^b^	<0.001
LSAS anxiety	37.98 ± 8.81^a^	55.26 ± 12.30^b^	43.18 ± 11.17^a^	64.38 ± 13.24^c^	<0.001
LSAS avoidance	40.00 (31.00–49.00)^a^	58.00 (49.00–68.00)^b^	46.00 (34.00–57.00)^a^	64.00 (59.00–78.00)^b^	<0.001

Values expressed as *n* (%), mean ± SD, or median (25th–75th percentiles) groups with different superscript letters were found to have statistically significant differences.

**Table 3:** Evaluation of Self-Esteem Scale among groups.

Variables	Control (*n* = 47)	Hirsutism-acnea (*n* = 35)	Infertility (*n* = 22)	Obesity (n = 29)	*P*
Self-esteem					
Low	1 (2.1)^a^	7 (20.0)^b^	4 (18.2)^b^	18 (62.1)^c^	<0.001
Medium	23 (48.9)^a^	25 (71.4)^b^	10 (45.5)^a^	8 (27.6)^a^	
High	23 (48.9)^a^	3 (8.6)^b^	8 (36.4)^a^	3 (10.3)^b^	
CSC					
Low	10 (21.3)	14 (40.0)	5 (22.7)	10 (34.5)	0.238
High	37 (78.7)	21 (60.0)	17 (77.3)	19 (65.5)	
TIP					
Low	3 (6.4)^a^	5 (14.3)^a^	1 (4.5)^a^	13 (44.8)^b^	<0.001
Medium	23 (48.9)^a^	18 (51.4)^a^	7 (31.8)^a^	9 (31.0)^a^	
High	21 (44.7)^abc^	12 (34.3)^c^	14 (63.6)^b^	7 (24.1)^ac^	
SC					
Low	34 (72.3)^a^	21 (60.0)^ab^	9 (40.9)^bc^	7 (24.1)^c^	<0.001
High	13 (27.7)^a^	14 (40.0)^ab^	13 (59.1)^bc^	22 (75.9)^c^	
DA					
None	29 (61.7)^a^	5 (14.3)^b^	0 (0.0)^b^	2 (6.9)^b^	<0.001
Low	15 (31.9)^a^	11 (31.4)^a^	3 (13.6)^a^	10 (34.5)^a^	
Medium	3 (6.4)^a^	12 (34.3)^b^	8 (36.4)^b^	10 (34.5)^b^	
High	0 (0.0)^a^	7 (20.0)^b^	11 (50.0)^c^	7 (24.1)^bc^	
DD					
Low	8 (17.0)^a^	6 (17.1)^a^	0 (0.0)^b^	12 (41.4)^c^	<0.001
Medium	20 (42.6)^ab^	22 (62.9)^b^	7 (31.8)^a^	12 (41.4)^ab^	
High	19 (40.4)^a^	7 (20.0)^b^	15 (68.2)^c^	5 (17.2)^b^	
PSS					
Low	38 (80.9)^a^	20 (57.1)^b^	1 (4.5)^c^	2 (6.9)^c^	<0.001
Medium	7 (14.9)^a^	14 (40.0)^b^	9 (40.9)^b^	11 (37.9)^b^	
High	2 (4.3)^a^	1 (2.9)^a^	12 (54.5)^b^	16 (55.2)^b^	
FTIR					
None	19 (40.4)	7 (20.0)	3 (13.6)	9 (31.0)	0.108
Medium	23 (48.9)	22 (62.9)	13 (59.1)	12 (41.4)	
High	5 (10.6)	6 (17.1)	6 (27.3)	8 (27.6)	
ID					
Low	13 (27.7)	8 (22.9)	4 (18.2)	13 (44.8)	0.124
Medium	21 (44.7)	20 (57.1)	8 (36.4)	10 (34.5)	
High	13 (27.7)	7 (20.0)	10 (45.4)	6 (20.7)	
PI					
Low	3 (6.4)	3 (8.6)	0 (0.0)	2 (6.9)	0.496
Medium	17 (36.2)	19 (54.3)	12 (54.5)	13 (44.8)	
High	27 (57.4)	13 (37.1)	10 (45.5)	14 (48.3)	
FR					
Low	1 (2.1)	2 (5.7)	1 (4.5)	1 (3.4)	0.603
Medium	19 (40.4)	20 (57.1)	9 (40.9)	11 (37.9)	
High	27 (57.4)	13 (37.1)	12 (54.5)	17 (58.6)	
PsI					
Low	37 (78.7)^a^	19 (54.3)^b^	5 (22.7)^c^	5 (17.2)^c^	<0.001
High	10 (21.3)^a^	16 (45.7)^b^	17 (77.3)^c^	24 (82.8)^c^	

Values are expressed as *n* (%), mean ± SD or median (25th–75th percentiles) groups with different superscript letters were found to have statistically significant differences.

**Table 4:** Evaluation of SF-36 scale among groups.

Variables	Control (*n* = 47)	Hirsutism-acnea (*n* = 35)	Infertility (*n* = 22)	Obesity (*n* = 29)	*P*
PF	88.00 (86.80–90.00)^a^	67.20 (64.00–73.80)^b^	74.90 (70.00–80.00)^b^	76.00 (70.50–80.20)^b^	<0.001
PRF	90.71 ± 1.94^a^	70.85 ± 7.39^b^	73.51 ± 7.98^b^	79.83 ± 5.39^c^	<0.001
Pain	89.00 (87.50–90.50)^a^	74.50 (69.90–81.00)^b^	75.00 (70.30–82.00)^b^	79.00 (75.00–82.00)^b^	<0.001
GH	74.00 (71.50–75.60)^a^	67.20 (58.00–71.40)^b^	66.30 (61.00–71.00)^b^	65.00 (61.00–68.00)^b^	<0.001
Vitality	71.00 (69.00–73.00)^a^	57.20 (51.00–64.40)^b^	55.00 (51.40–61.00)^b^	59.00 (55.60–61.80)^b^	<0.001
SF	95.50 (93.00–97.00)^a^	76.00 (66.80–87.00)^b^	80.10 (76.00–84.80)^b^	78.40 (76.00–84.00)^b^	<0.001
ERF	95.10 ± 2.31^a^	75.17 ± 10.66^b^	79.01 ± 8.77^b^	79.63 ± 7.97^b^	<0.001
EWB	79.82 ± 4.15^a^	58.20 ± 8.92^b^	60.97 ± 6.87^b^	66.94 ± 5.57^c^	<0.001

Values expressed as *n* (%), mean ± SD, or median (25th–75th percentiles) groups with different superscript letters were found to have statistically significant differences.
